# The Long-Term Evolution of Macular Retinoschisis in Eyes with Myopic Choroidal Neovascularization Treated with Anti-Vascular Endothelial Growth Factors

**DOI:** 10.3390/jcm15145475

**Published:** 2026-07-13

**Authors:** Han-Hao Tsai, Fang-Ting Chen

**Affiliations:** 1 Department of Ophthalmology, China Medical University Hospital, Taichung 404, Taiwan; u102001016pro@gmail.com; 2 Department of Ophthalmology, Far Eastern Memorial Hospital, New Taipei City 220, Taiwan; 3 Department of Ophthalmology, National Taiwan University Hospital, Taipei 100, Taiwan

**Keywords:** pathologic myopia, myopic choroidal neovascularization (mCNV), anti-vascular endothelial growth factor (anti-VEGF), macular retinoschisis (MRS)

## Abstract

**Background:** To investigate the structural changes and visual outcomes in eyes with myopic choroidal neovascularization (mCNV) treated with anti-vascular endothelial growth factor (anti-VEGF) compared with fellow eyes over a long-term follow-up period. **Methods:** A retrospective paired-eye cohort study was conducted, and 51 patients (80.4% female, mean age 51.8 years) with an average follow-up duration of 32.8 months were enrolled. The study included patients with active, treatment-naïve mCNV in one eye and a fellow eye without mCNV. Final visual outcomes and macular retinoschisis (MRS) progression were compared between mCNV eyes treated with intravitreal anti-VEGF injections and fellow eyes. Baseline parameters potentially associated with MRS progression in mCNV eyes were analysed. **Results:** The final VA increased in mCNV eyes but remained worse than that in fellow eyes (logMAR 0.3 vs. 0.16, *p* = 0.037). The percentages of treated mCNV eyes and fellow eyes showing MRS progression were similar (18.6% vs. 16.3%, *p* = 0.782). When mCNV eyes with and without MRS progression were compared (*n* = 10 and 41, respectively), MRS at baseline was the only significant predictor of MRS progression (70% vs. 17.1%, *p* = 0.002). A greater percentage of eyes with MRS progression exhibited higher vitreomacular interface (VMI) grading at baseline, although the difference was not statistically significant (40% vs. 14.7%; *p* = 0.083). **Conclusions:** Anti-VEGF treatment in mCNV eyes did not increase the incidence of MRS compared with fellow eyes or aggravate MRS. However, the presence of MRS at baseline may increase the risk of MRS progression after treatment, and close monitoring of this subgroup is warranted.

## 1. Introduction

Pathologic myopia is a progressively degenerative eye disease with the potential to lead to irreversible blindness, which frequently hinders the productivity of working-age patients. The incidence of pathologic myopia is increasing worldwide, and the reported prevalence is 1% in Caucasians and 1~3% in Asians [1]. In Taiwan, the prevalence of high myopia is much higher than that in most other Asian populations [2]. The formation of posterior staphyloma, along with complex vitreomacular adhesion, leads to many sight-threatening macular complications, including chorioretinal (CR) atrophy, macular retinoschisis (MRS) and other myopic traction maculopathies, and choroidal neovascularization [3].

Myopic choroidal neovascularization (mCNV), characterized by the growth of abnormal choroidal vessels through defects in Bruch’s membrane caused by myopic degenerative changes, is one of the most vision-threatening complications of pathologic myopia [4]. It develops at a rate of 5.2% to 11.3% in patients with pathologic myopia [5,6]. Without timely treatment, mCNV may lead to subretinal fibrosis, chorioretinal atrophy, and irreversible central vision loss; therefore, anti-vascular endothelial growth factor (VEGF) therapy has become the first-line treatment because of its proven efficacy and safety [4,7,8,9].

However, an increase in the incidence of MRS was observed in some studies after the intravitreal injection (IVI) of anti-VEGF [10,11]. MRS, characterized by splitting of the retinal layers within the macula and considered a key manifestation of myopic traction maculopathy, is another major cause of visual decline in myopic patients, with a prevalence of approximately 9% to 27.9% [12,13,14]. Vitreomacular traction (VMT) is believed to play an important role in the development of MRS on the basis of the observation of MRS resolution following the release of VMT, either spontaneously or surgically [15,16]. A retrospective study demonstrated the natural course of MRS, with 11.6% of patients showing progression, 84.5% showing stability and 3.9% showing improvement over a mean follow-up of 36.2 months [17]. Among the anti-VEGF-treated mCNV eyes, the cumulative rates of MRS progression and onset were 15.2% at 3 months, 19.3% at 6 months, 23.4% at 1 year, 28.5% at 2 years, 29.2% at 3 years, and 32.2% at 5 years in Tsui et al.’s study [18]. The reason for the potential worsening effect of anti-VEGF treatment over the course of MRS is not clear, but the proposed underlying mechanisms include shrinkage of the mCNV membrane and the vitreous disturbance caused by repeated IVIs [19]. Although a recent study concluded that MRS progression was not associated with the frequency of intravitreal conbercept injections [20], long-term follow-up data and comparisons with matched non-mCNV eyes are still lacking.

Thus, we conducted a study to review the long-term evolution of MRS in mCNV eyes treated with IVI of anti-VEGF compared with non-mCNV fellow eyes. In addition, we aimed to identify possible risk factors associated with MRS progression in treated mCNV eyes.

## 2. Methods

In this retrospective paired-eye cohort study, the evolution of MRS in mCNV eyes and in fellow eyes over 12 months of follow-up was assessed. The study was performed in accordance with the guidelines of the Declaration of Helsinki and was approved by the institution’s ethical review commission (FEMH No. 111164-E).

We collected data from patients who were eligible for reimbursement for anti-VEGF agents for mCNV by the National Health Insurance in Taiwan. The patients were diagnosed with mCNV, which was treated via IVI of aflibercept (Eylea, Regeneron, Tarrytown, NY, USA), ranibizumab (Lucentis, Novartis, Basel, Switzerland) or bevacizumab (Avastin, Genentech, San Francisco, CA, USA) on a pros re nata basis at Far-Eastern Memorial Hospital, Banqiao, New Taipei City, Taiwan, from July 2016 to July 2020. Retreatment was performed according to disease activity, primarily based on OCT findings (persistent or recurrent intraretinal or subretinal fluid), new retinal hemorrhage, and deterioration of VA attributable to active mCNV. All IVIs were performed under topical anesthesia using standard sterile techniques through the pars plana (3.5–3.75 mm posterior to the limbus) with a 30-gauge needle. The inclusion criteria were as follows: (1) active and treatment-naïve mCNV in one eye and no evidence of mCNV in the fellow eye, (2) both eyes with high myopia, defined as a spherical equivalent < −6.0 D or axial length ≥ 26 mm, and (3) follow-up duration ≥ 12 months. Participants were not included if (1) coexisting nonmyopic maculopathies were present, (2) subfoveal fibrosis or macular atrophy was present, (3) the status of mCNV remained active at the last follow-up visit, or (4) new onset of mCNV in the fellow eye occurred within 12 months. The study duration ranged from the time of mCNV diagnosis to the time of the last visit. For patients with new onset of mCNV in their fellow eyes, the study end point was set at the last visit date before the new onset of mCNV.

Every patient received a complete ocular examination, including slit-lamp biomicroscopy, indirect ophthalmoscopy, best-corrected visual acuity (BCVA), refractive error, axial length measurement with an optical biometer (LENSTAR LS 900, Haag-Streit USA, Mason, OH, USA), colour fundus photography (TRC-NW8, TOPCON, Tokyo, Japan), spectral domain optical coherence tomography (SD-OCT; RTVue-100, Optovue, Inc., Fremont, CA, USA before 2017; Zeiss Cirrus HD-OCT 5000, Carl Zeiss Meditec, Dublin, CA, USA after 2017), and fluorescein angiography (FA; Spectralis HRA, Heidelberg Retina Angiograph, Heidelberg Engineering, Heidelberg, Germany).

Through colour fundus photography, we classified the severity of CR atrophy at the macula into 4 grades: (1) Grade 1: tessellated fundus, (2) Grade 2: diffuse CR atrophy, (3) Grade 3: patchy CR atrophy, and (4) Grade 4: macular atrophy, with reference to the international photographic classification and grading system for myopic maculopathy [21]. The vitreomacular interface (VMI) status and the extent of MRS were evaluated through horizontal and vertical 6 mm raster scans via SD-OCT. MRS was defined as the splitting of the inner and/or outer retinal layers at macula, resulting in hyporeflective intraretinal cystoid spaces on OCT [22,23].

The status of the VMI was classified into 3 grades, which were positively correlated with traction force in order: (1) Grade 1: no VMT (the posterior hyaloid membrane was completely adherent to or completely separated from the macula), (2) Grade 2: broad VMT (the posterior hyaloid membrane was partially separated from the macula with a >1.5 mm adhesion area), and (3) Grade 3: focal VMT (<1.5 mm adhesion area) [24]. Other related findings on SD-OCT, including foveal detachment (FD), epiretinal membrane (ERM), a lamellar macular hole (LMH), a full-thickness macular hole (FTMH), macular hole retinal detachment (MHRD), and a dome-shaped macula (DSM), were also recorded. The size of the mCNV area was calculated with the built-in program of the Spectralis HRA.

**Figure 1:**
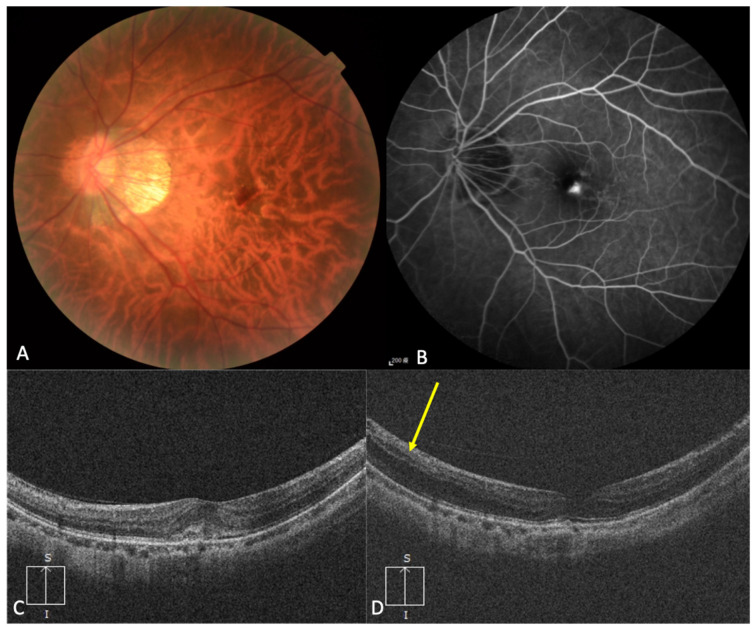
Representative case of new-onset MRS during follow-up. (**A**) Color fundus photograph showing pathologic myopia with mCNV. (**B**) Fluorescein angiography demonstrating leakage from active mCNV. (**C**) Baseline OCT obtained at the time of mCNV diagnosis showing no evidence of MRS. (**D**) Follow-up OCT demonstrating regressed mCNV with newly developed outer MRS in the inferior macula after 2 years and 7 months of follow-up (yellow arrow).

**Figure 2:**
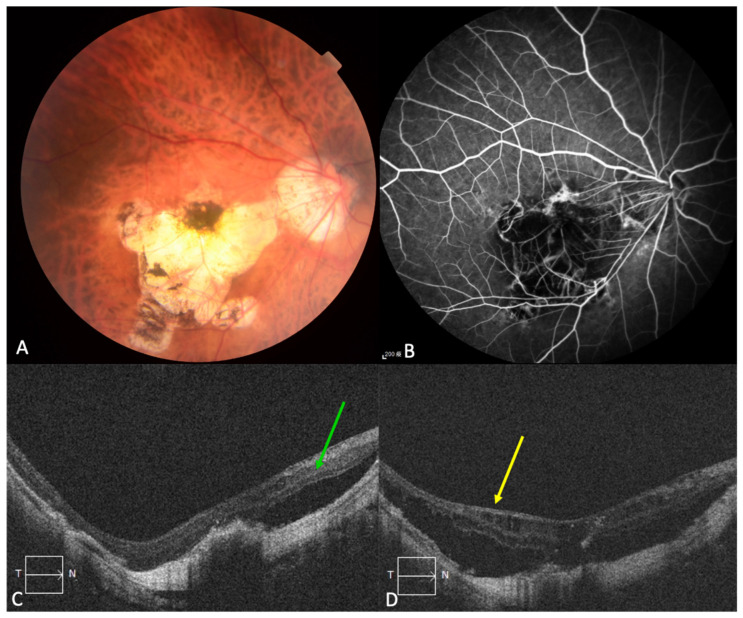
Representative case of expansion of pre-existing MRS during follow-up. (**A**) Color fundus photograph showing pathologic myopia with mCNV and patchy chorioretinal atrophy. (**B**) Fluorescein angiography demonstrating leakage from active mCNV. (**C**) Baseline OCT showing pre-existing MRS involving the nasal macula (green arrow). (**D**) Follow-up OCT demonstrating expansion of both inner and outer MRS toward the temporal macula (yellow arrow), with increased extent and height compared with baseline, accompanied by regression of the mCNV lesion after 2 years and 6 months of follow-up.

The progression of VMI status was defined as an increased VMI grade at the last visit compared with baseline. The progression of MRS was defined as the new onset of MRS in eyes without baseline MRS, or the expansion of pre-existing retinoschisis (in height and/or horizontal extent) with baseline MRS or the development of a LMH, FTMH, or FD [17]. Representative OCT images demonstrating MRS progression are shown in Figure 1 and Figure 2.

The data were analysed with R software for macOS (version 4.1.2, R Foundation for Statistical Computing, Vienna, Austria). The normality of continuous variables was assessed using the Shapiro–Wilk test. Paired comparisons between mCNV eyes and fellow eyes were performed using paired t-tests or Wilcoxon signed-rank tests, as appropriate. Comparisons between independent groups, including MRS progression and non-progression eyes, were performed using independent t-tests or Wilcoxon rank-sum tests, as appropriate. McNemar’s test was used for paired categorical variables, whereas Pearson’s χ^2^ test or Fisher’s exact test was used for independent categorical variables. A two-sided *p* value < 0.05 was considered statistically significant.

## 3. Results

Overall, 51 patients were enrolled in this study, with 80.4% of patients being female. The mean age was 51.8 ± 11.1 years, with a range of 29 to 77 years. Among a total of 135 injections, ranibizumab accounted for 80 (59.3%), aflibercept accounted for 48 (35.6%), and bevacizumab accounted for 7 (5.2%). The median number of IVIs was 2 (interquartile range, 1–3). The mean duration of follow-up was 32.8 ± 12.4 months (range: 12–59 months).

The baseline characteristics of the mCNV eyes and fellow eyes are presented in Table 1. A poorer baseline VA (logMAR) (0.7 vs. 0.22, *p* < 0.001) and greater central foveal thickness (CFT) (290 vs. 255 μm, *p* < 0.001) were observed in mCNV eyes than in fellow eyes. There was no statistically significant difference in refractive error, axial length, CR atrophy grade, VMI grade, or the presence of MRS or other macular structural pathology at baseline between mCNV eyes and fellow eyes.

The final outcomes in the mCNV eyes and fellow eyes are presented in Table 2. At the final visit, the progression rates of VMI status were 14% and 7% in the mCNV eyes and fellow eyes, respectively (*p* = 0.317). There was no significant difference in the percentage of treated mCNV eyes and fellow eyes showing MRS progression (18.6% vs. 16.3%, *p* = 0.782). Among eyes with MRS progression, MRS progression mainly originated from the expansion of the original MRS rather than new-onset MRS. Other retinal complications associated with high myopia, including LMHs, FTMHs and RRD, were rare in both mCNV eyes and fellow eyes. Two fellow eyes showed new onset of mCNV during follow-up. With respect to visual outcomes, while the final BCVA (logMAR) in the treated mCNV eyes increased, it remained inferior to that in the fellow eyes (0.3 vs. 0.16, *p* = 0.037). The fellow eyes showed stable visual acuity during the follow-up period.

The MRS progression rate in all mCNV eyes was 19.6% (10/51). To identify any possible risk factors for MRS progression in mCNV eyes, we compared the baseline characteristics between MRS progression and nonprogression eyes (Table 3). There was no significant difference in axial length, CNV size, baseline CR atrophy grade, or VMI status between MRS progression and nonprogression eyes. The percentage of baseline ERM or DSM was not significantly different between MRS progression and nonprogression eyes. The only parameter that was significantly different at baseline was the presence of MRS. The percentage of MRS at baseline was much greater in MRS progression eyes than in nonprogression eyes (70% vs. 17.1%, *p* = 0.002). Notably, MRS progression eyes had higher baseline VMI grades than MRS nonprogression eyes did, although the difference was not statistically significant (Grade 1: 60% vs. 85.4%; Grade 2: 30% vs. 9.8%; Grade 3: 10% vs. 4.9%; *p* = 0.083).

The final outcomes of MRS progression and nonprogression eyes after anti-VEGF treatment for mCNV are presented in Table 4. Comparison of these outcomes revealed that there was no significant difference in the number of IVIs or the percentage of eyes with VMI progression between the groups. Visual acuity was poorer in MRS progression eyes than in MRS nonprogression eyes; however, the difference was not statistically significant (logMAR 0.8 vs. 0.22; *p* = 0.123).

## 4. Discussion

In this retrospective study, we analysed patients with anti-VEGF-treated mCNV in one eye and no mCNV in the fellow eye with a mean follow-up duration of 32.8 months. We found no significant difference in the percentage of treated mCNV eyes and fellow eyes showing MRS progression (18.6% vs. 16.3%, *p* = 0.782). The percentage of mCNV eyes and fellow eyes showing VMI progression was also not significantly different (14% vs. 7%, *p* = 0.317). In mCNV eyes, there were no other retinal complications except for one eye in which an FTMH developed during follow-up. These findings suggest that IVI of anti-VEGF agents does not induce significant structural changes, such as MRS or VMI, in most mCNV eyes. This result coincided with that of the post hoc analysis by Zhou et al. [20], who reported that the number of intravitreal conbercept injections was not an independent risk factor for the progression or new onset of MRS at the 9-month follow-up (odds ratio = 0.996, *p* = 0.982). In general, anti-VEGF treatment for mCNV may not induce additional structural insult to the macula compared with fellow eyes.

In the present study, the only significant risk factor for MRS progression in treated mCNV eyes was the presence of MRS at baseline. MRS at baseline was found in 70% of the progression eyes and 17.1% of the nonprogression eyes (*p* = 0.002). In Tsui et al.’s study, 35 out of 216 eyes (16.2%) without MRS at baseline developed MRS, whereas 60 out of 79 eyes (75.9%) with MRS at baseline showed MRS progression, which is consistent with our findings. Similar to our study, MRS at baseline was strongly associated with MRS progression, and outer retinoschisis was identified as a significant risk factor associated with progression or new onset of MRS after anti-VEGF treatment (β = 8.586, *p* = 0.003) [18]. The study surveying the natural course of MRS also supports the correlation of baseline MRS severity and the risk of MRS progression. Shimada et al. reported an overall progression rate of 11.6% during a mean follow-up of 36.2 months, with progression rates increasing from 6.3% (S0), 3.6% (S1), 8.9% (S2), and 13.0% (S3) to 42.9% (S4) [17]. This stage-dependent increase in progression rate indicates that baseline MRS severity reflects the underlying structural vulnerability and is an important predictor of subsequent MRS progression.

The relationship between baseline VMI status and MRS progression in treated mCNV eyes remains a matter of debate. One study revealed that the baseline vitreoretinal adhesion rate was significantly greater in patients with progression or new-onset MRS than in those without (*p* = 0.016) [20]. However, another study did not identify baseline VMT as a risk factor for MRS progression [18]. In our study, eyes with MRS progression had higher VMI grades than did nonprogression eyes (Grade 3: 10% vs. 4.9%; Grade 2: 30% vs. 9.8%; Grade 1: 60% vs. 85.4%; *p* = 0.083), although the difference was not statistically significant. Baseline VMT, as an anterior–posterior traction force to the macula, theoretically contributes to the progression of MRS in highly myopic eyes. However, further investigation is needed to determine whether baseline VMT is a definite risk factor for MRS progression in treated mCNV eyes.

Several possible mechanisms for how IVI of anti-VEGF affects MRS progression in mCNV eyes have been proposed in previous studies. Some researchers have proposed that the injection procedure may have a direct mechanical effect on the vitreous body, which in turn leads to macular traction [25,26]. In addition, rapid influx of the drug could lead to vitreous perturbation and subsequent vitreous liquefaction, forming an inwards force to the retinal surface [20]. The shrinkage of fibrovascular tissue induced by anti-VEGF agents is also believed to be a cause of retinal tissue separation [19]. In our study, the change in VMI status did not differ between mCNV eyes and fellow eyes during follow-up (percentage of eyes with progression of VMI status: 14% vs. 7%; *p* = 0.317), indicating that IVI of anti-VEGF did not significantly affect the vitreous status. The effect of fibrovascular tissue shrinkage seemed subtle in our study, as there was no significant difference in CNV size (mean 320 μm^2^ vs. 280 μm^2^, *p* = 0.661) between MRS progression and nonprogression eyes. According to our study results, the presence of MRS at baseline was the major risk factor for MRS progression in mCNV eyes. Therefore, eyes with compromised macular integrity and decreased structural stability may be more likely to be affected by anti-VEGF treatment than those without.

Our study demonstrated the long-term effectiveness of anti-VEGF treatment in mCNV eyes, and the functional results were comparable to those of several previous studies [9,27,28,29]. We found that mCNV eyes treated with anti-VEGF agents achieved a significant improvement in BCVA (from logMAR 0.69 to 0.52, *p* = 0.003) with a median of 2 injections over a median of 32.8 months of follow-up. Despite successful treatment, the final BCVA in the mCNV eyes remained inferior to that in the fellow eyes (logMAR 0.3 vs. 0.16, *p* = 0.037). With respect to the influence of MRS progression on final visual outcome in treated mCNV eyes, our study revealed poorer visual outcomes in eyes with MRS progression than in those without MRS progression [VA improvement (+5 letters vs. +18 letters, *p* = 0.264), final BCVA (logMAR 0.8 vs. 0.22, *p* = 0.123)], although the difference was not statistically significant, probably because of an insufficient number of cases in the MRS progression group.

There are several limitations in our study. First, the retrospective design and the low incidence of MRS progression limited the number of cases in the progression group, leading to a small sample size and low treatment exposure (median of two injections). As a result, the study may be underpowered to detect any effect of IVI on MRS progression. Furthermore, serial OCT images were taken by different machines (RTVue-100 before 2017; Cirrus HD-OCT 5000 after 2017) in some patients, and the variability between machines was not adjusted. Nevertheless, the measured retinal thickness may be relatively comparable between the 2 machines [30]. In mCNV eyes, it may be unclear whether the occurrence of FD was due to MRS progression or the exudation of mCNV. We attributed FD to MRS progression if there was no evidence of mCNV activity (i.e., fuzzy border of the subretinal hyperdense lesion on OCT and/or accompanied macular haemorrhage). However, further investigation is needed to determine whether FD results from MRS progression, mCNV exudation, or a combination of these factors. Finally, MRS progression was assessed using predefined qualitative OCT criteria rather than quantitative measurements of schisis height or area [17]. Therefore, subtle changes in MRS severity could not be quantified, and the extent of MRS progression could be undetected in such conditions. Future studies in which a classification system for MRS severity is incorporated or height and area are precisely quantified could help differentiate high-risk MRS from stable MRS in mCNV eyes. Identifying high-risk eyes may facilitate more tailored management and careful consideration when administering anti-VEGF treatment.

## 5. Conclusions

In mCNV eyes treated with anti-VEGF, there was no detrimental effect on the evolution of MRS compared with that in fellow eyes over a mean follow-up of 32.8 months. The macular structures remained stable in most treated mCNV eyes, with extremely few retinal complications and significantly improved vision. However, in eyes with concurrent MRS at baseline, there may be a greater risk of MRS progression and worse visual outcomes after anti-VEGF treatment than in eyes without MRS at baseline. Close monitoring of MRS evolution in these eyes is mandatory.

## Figures and Tables

**Table 1:** Baseline characteristics of mCNV eyes and fellow eyes.

Characteristics	mCNV Eyes (*n* = 49) ^a^	Fellow Eyes (*n* = 49) ^a^	*p* Value
Baseline BCVA (LogMAR) [Median (IQ)]	0.7 (0.4–1)	0.22 (0.1–0.4)	<0.001 ***
Refraction (Mean ± SD) ^b^	−13 ± 4	−12.9 ± 5	0.886
Axial length (mm)	29.3 ± 1.5	29.2 ± 1.6	0.607
CFT (μm)	290 (251–320)	255 (244–270)	<0.001 ***
CR atrophy (Grade 1/2/3/4) (%) ^c^	24/9/6/1 (60/22.5/15/2.5)	28/5/5/2 (70/12.5/12.5/5)	0.585
VMI (Grade 1/2/3) (%)	39/7/3 (79.6/14.3/6.1)	45/3/1 (91.8/6.1/2)	0.115
MRS [*n* (%)]	14 (28.6)	12 (24.5)	0.617
ERM [*n* (%)]	12 (24.5)	9 (18.4)	0.317
LMH [*n* (%)]	1 (2)	1 (2)	1
DSM [*n* (%)]	16 (32.7)	11 (22.5)	0.197

mCNV: Myopic choroidal neovascularization; BCVA: Best-corrected visual acuity; CFT: Central foveal thickness; CR: Chorioretinal; VMI: Vitreomacular interface; MRS: Macular retinoschisis; N/A: not applicable; ERM: Epiretinal membrane; LMH: Lamellar macular hole; DSM: Dome-shaped macula. ^a^ Excluded 2 eyes without baseline OCT data in the fellow eyes; ^b^ 13 eyes were excluded due to corrected refraction by cataract or refractive surgery; ^c^ excluded 9 eyes for lack of color fundus photograph of fellow eyes. *** *p*-value < 0.001.

**Table 2:** Final outcomes of mCNV eyes and fellow eyes.

Characteristics	mCNV Eyes (*n* = 43) ^a^	Fellow Eyes (*n* = 43) ^a^	*p* Value
VMI progression [*n* (%)]	6 (14)	3 (7)	0.317
MRS progression [*n* (%)]	8 (18.6)	7 (16.3)	0.782
Newly onset MRS [*n* (%)]	2 (4.7)	1 (2.3)	0.564
Newly onset FD [*n* (%)]	1 (2.3)	1 (2.3)	1
Expansion of original MRS [*n* (%)]	6 (14.0)	6 (14.0)	1
Newly onset of other complications			
LMH [*n* (%)]	0 (0)	1 (2.3)	N/A
FTMH [*n* (%)]	1 (2.3)	0 (0)	N/A
MHRD [*n* (%)]	0 (0)	0 (0)	N/A
RRD [*n* (%)]	0 (0)	1 (2.3)	N/A
Newly formed mCNV [*n* (%)]	N/A	2 (4.7)	N/A
Final CFT (μm) [Median (IQ)]	244 (225–264)	258 (241–272)	0.134
Final BCVA (LogMAR) ^b^	0.3 (0.22–0.77)	0.16 (0.05–0.4)	0.037 *
BCVA change (LogMAR) ^b^	−0.12 (−0.48–0.05)	0 (−0.12–0.1)	0.037 *

mCNV: Myopic choroidal neovascularization; VMI: Vitreomacular interface; MRS: Macular retinoschisis; FD: foveal detachment; LMH: Lamellar macular hole; FTMH: Full-thickness macular hole; MHRD: Macular hole with retinal detachment; RRD: Rhegmatogenous retinal detachment; N/A: not applicable; CFT: Central foveal thickness; BCVA: Best-corrected visual acuity. ^a^ Excluded 8 patients without final OCT data for fellow eyes. ^b^ Excluded 1 patient without final BCVA in mCNV eye. * *p*-value < 0.05.

**Table 3:** Baseline characteristics of MRS progression vs. MRS non-progression mCNV eyes.

Baseline Characteristics	MRS Progression Eyes (*n* = 10)	Non-Progression Eyes (*n* = 41)	*p* Value
Age (mean ± SD)	51.2 ± 12.5	51.9 ± 10.9	0.872
Baseline BCVA (LogMAR) [Median (IQ)]	0.7 (0.57–1)	0.52 (0.3–0.8)	0.109
Refraction (D) ^a^	−12.75 ± 3.2	−13.04 ± 4.1	0.842
Axial length (mm)	29.48	29.25	0.664
CNV size (μm^2^)	320 (165–683)	280 (120–530)	0.661
CR atrophy (Grade 1/2/3/4) (%)	6/2/2/0 (60/20/20/0)	23/11/5/2 (56.1/26.8/12.2/4.9)	0.884
VMI (Grade 1/2/3) (%)	6/3/1 (60/30/10)	35/4/2 (85.4/9.8/4.9)	0.083
MRS [*n* (%)]	7 (70)	7 (17.1)	0.002 **
FD [*n* (%)]	0 (0)	3 (7.3)	1
ERM [*n* (%)]	4 (40)	8 (19.5)	0.218
DSM [*n* (%)]	4 (40)	13 (31.7)	0.714
Baseline CFT (μm)	302 (267–328)	286 (245–312)	0.275

MRS: Macular retinoschisis; BCVA: Best-corrected visual acuity; D: Diopter; CNV: Choroidal neovascularization; CR: Chorioretinal; VMI: Vitreomacular interface; FD: Foveal detachment; ERM: Epiretinal membrane; DSM: Dome-shaped macula; CFT: Central foveal thickness. ^a^ 13 eyes were excluded due to corrected refraction by cataract or refractive surgery. ** *p*-value < 0.005.

**Table 4:** Final outcomes of MRS progression vs. MRS non-progression mCNV eyes.

Characteristics	MRS Progression Eyes (*n* = 10)	Non-Progression Eyes (*n* = 41)	*p* Value
IVI numbers [Median (IQ)]	3 (2–3.75)	2 (1–3)	0.317
VMI progression [*n* (%)]	3 (30)	4 (9.8)	0.126
Final CFT (μm)	265 (242–289)	245 (222–261)	0.102
Final BCVA (LogMAR)	0.8 (0.24–1.23)	0.22 (0.22–0.52) ^a^	0.123
BCVA change (LogMAR)	−0.05 (−0.28–0.25)	−0.18 (−0.48–0) ^a^	0.264

MRS: Macular retinoschisis; mCNV: myopic choroidal neovascularization; IVI: Intravitreal injection; VMI: Vitreomacular interface; CFT: Central foveal thickness; BCVA: Best-corrected visual acuity. ^a^ Excluded 1 patient without final BCVA in mCNV eye.

## Data Availability

The datasets generated and/or analysed during the current study are not publicly available due to the restriction by IRB, but are available from the corresponding author on reasonable request.

## Abbreviations

mCNV	Myopic choroidal neovascularization
anti-VEGF	Anti-vascular endothelial growth factor
MRS	Macular retinoschisis
VMI	Vitreomacular interface
CR	Chorioretinal
IVI	Intravitreal injection
VMT	Vitreomacular traction
BCVA	Best-corrected visual acuity
FD	Foveal detachment
ERM	Epiretinal membrane
LMH	Lamellar macular hole
FTMH	Full-thickness macular hole
MHRD	Macular hole retinal detachment
DSM	Dome-shaped macula
CFT	Central foveal thickness
